# Effect of commonly used cosmetic preservatives on skin resident microflora dynamics

**DOI:** 10.1038/s41598-021-88072-3

**Published:** 2021-04-22

**Authors:** Daniela Pinto, Tiziana Ciardiello, Matteo Franzoni, Francesca Pasini, Giammaria Giuliani, Fabio Rinaldi

**Affiliations:** Human Advanced Microbiome Project-HMAP, Milan, Italy

**Keywords:** Cell biology, Microbiology, Health care

## Abstract

Human skin is populated by various microorganisms, the so-called microbiota, such as bacteria, viruses, yeasts, fungi, and archaea. The skin microbiota is in constant contact with the surrounding environment which can alter its eubiotic state. Recently it has been also observed that the application of cosmetic products can alter the balance of the skin microbiota. This effect may be attributed to many factors including the residual activity of the preservatives on the skin. In the present work, we studied the effect of eleven preservatives commonly found in cosmetic products on *Propionibacterium acnes*,* Staphylococcus epidermidis*, and *Staphylococcus aureus *in vitro using 3D skin models and culture-dependent methods. Also, the effect on Histone deacetylase 3 (HDAC3) has been investigated. Among tested combinations, three resulted as the best suitable for restoring a pre-existing dysbiosis since they act moderately inhibiting *C. acnes* and strongly *S. aureus* without simultaneously inhibiting the growth of *S. epidermidis*. The other four combinations resulted as the best suitable for use in topical products for skin and scalp in which it is necessary to preserve the eubiosis of the microbiota. Some of the tested were also able to increase HDAC3 expression. Taking together these data highlight the role of preservatives of skin resident microflora dynamics and could provide a reference for correctly choice preservatives and dosage in cosmetic formulations to preserve or restore homeostasis of skin microbiota.

## Introduction

Human skin acts as the first line of defense against infectious and toxic external agents. It is populated by various microorganisms, the so-called microbiota, such as bacteria, viruses, yeasts, fungi, and archaea. They have been well characterized and classified^1^ and are distributed in a complex and well-defined balance^2,3^.

In recent years, the microbiome, and especially the skin microbiome, has been reported to play an important role in human health. Indeed, the large population of microbes that lives on the skin in addition to human innate immunity creates the skin barrier that allows maintaining the skin healthy^4^. When the balance of the skin microbiota is disturbed, there is an alteration of the diversity and population of the skin microbiota with a transition from eubiosis to dysbiosis. In these cases, the individual's skin becomes more susceptible to the action of external agents and there is an increased incidence of several cutaneous diseases like inflammatory dermatosis and cutaneous infection^5^.

The skin microbiota, as our most outer skin layer, is costantly contact with the surrounding environment (UV radiation, frost, pollution, or disinfectants).

As a consequence of changes in both the external and internal environment, the skin can respond rapidly with neural signals or slowly with humoral or immune signals^6^. All these signals act in coordination under the control of the neuroendocrine system^7^.

Indeed, similarly to the gut, skin is also to be considered a neuroendocrine organ^6^ in constant interaction with both the environment^6^ and its inhabiting microbiota^8^. The skin neuroendocrine system preserves and maintains skin integrity, functionality, and homeostasis^7^. Indeed, skin can release neuropeptides and neurohormones such as, for example, substance P, calcitonin gene‐related peptide, and catecholamines that can directly affect the behavior of common skin‐associated bacteria. They can also spread in the extracellular matrix^9^ and sweat^10^ changing their concentration in relation to the host neurophysiological activity or disorders.

At the same time, skin microbiota can synthesize and release molecules and neurohormones-like compounds (e.g. histamine, glutamate, and γ‐aminobutyric acid, etc.) which can interact with skin physiology (dialog between).

By its interaction with the different cutaneous hormones and neurohormones, the skin microbiota could be considered an intrinsic factor of cutaneous homeostasis and an essential component of the barrier to be preserved in order to fight against environmental and, most in general, external aggressions^11,12^.

There is growing evidence that pollutants, phthalates, and other chemicals, also that included in cosmetic products can act as endocrine disruptors, interfering with the skin neuroendocrine system.

It has also recently been observed that the application of cosmetic products to the skin can alter the balance of the skin microbiota, compromising the eubiosis of the skin, mucous membranes, and the scalp. Indeed, the topical application of cosmetic products such as hygiene products, moisturizers, anti-aging oily products, soaps, shampoos, lotions can alter the lipid film that covers the skin and affect the diversity of the skin's resident microflora^13,14^.

This negative process may be attributed to many factors including the residual activity of the preservatives on the skin since they remain active in products after these have been applied to the skin. Indeed, the preservatives can interact with the microbes present in this environment changing the amount of the balance of the bacterial population^13^. The influence of cosmetic preservatives on skin resident bacteria still requires further investigations.

To understand the effect of most frequently used preservatives on skin microflora, a 3D skin models specifically developed for studying interactions between the skin and its microbiome was used^15^.

The effect of eleven preservatives commonly found in cosmetic products on *Propionibacterium acnes*,* Staphylococcus epidermidis*, and *Staphylococcus aureus* was investigated in vitro using culture-dependent methods. Also the effect on Histone deacetylase 3 (HDAC3), a key mediator in orchestrating commensal bacteria-dependent intestinal and skin has been investigated^16,17^.

## Materials and methods

### Materials

Reinforced clostridium medium broth (RCM), Mannitol salt agar (MS), Mueller–Hinton broth (MH), and Agar base were purchased from BIOTEC (Grossetto, GR, Italy). Furazolidone was purchased from Sigma-Aldrich (Milan, Mi, Italy).

The following preservatives were tested in different combinations: sodium benzoate, phenoxyethanol, ethylhexylglycerin, gluconolactone, hydroxyacetophenone, phenylpropanol, propanediol, caprylyl glycol, tocopherol, sodium anisate, 1,2-hexanediol, tetrasodium glutamate diacetate, benzyl alcohol, benzoic acid, dehydroacetic acid, o-cymen-5-ol, ppg-3 benzyl ether myristate, tropolone, levulinic acid, sodium levulinate, ammonium acryloyldimethyltaurate/vp copolymer, potassium sorbate.

Since the present work aims to study the commonest used preservative in cosmetic products, we decided to test them as they are usually used in terms of combination and suggested % of usage. This according to the cosmetic practice in which a preservative is never used alone as it would not be able to have the preservative efficacy required to pass the challenge test.

All the tested formulations were prepared by dispersing or solubilizing preservatives in water, by stirring.

Ammonium acryloyldimethyltaurate/vp copolymer (Aristoflex AVC) was then incorporated with a Silverson emulsifier (Crami Group Srl, MI, Italy). Aristoflex AVC was added at different percentages to obtain formulations with similar viscosity. As the gel was obtained, pH was regulated to achieve the maximum preservative efficacy.

The Histone deacetylase 3 (HDAC3) and Glyceraldehyde-3-Phosphate Dehydrogenase (GAPDH) TaqMan™ Gene Expression Assays (FAM) were purchased from Life Technologies (Milan, Italy).

### Bacterial strains

*Staphylococcus aureus subsp. aureus (S. aureus)* ATCC-25923, *Staphylococcus epidermidis (S. epidermidis)* ATCC-14990, and *Cutibacterium acnes (C. acnes)* ATCC-11827 were purchased from American Type Culture Collection (ATCC) (LGC Standards S.r.l., Sesto San Giovanni, MI, Italy). *Staphylococcus aureus* and *S. epidermidis* were aerobically cultured in MH or MS for 20- 24 h. *Cutibacterium acnes* were anaerobically cultured in RCM for 20- 24 h.

### Full-thikness skin model

Labskin 3D (Innovenn Ltd.)^15^ was used as a reconstructed skin model. The tissues were handled according to the instructions of the manufacturer. Immediately after arriving each insert was transferred from the delivery plate into a 12 well plate filled with approximately 4–5 ml of preheated Labskin maintenance medium. Inserts were then incubated overnight at 37 °C, with 5% CO_2_ before being inoculated by skin commensal bacteria.

### In vitro study of the influence of preservatives on skin microflora

Cultures of *S. aureus* ATCC-25923, *S. epidermidis* ATCC-14990, and *C. acnes* ATCC-11827 in the respective media at late exponential phase of growth were used to prepare a microbial suspension in GS-24 sterile medium. The inoculum density was obtained by dilution of the culture to OD600nm = 0.25 (10^8^ CFUmL^−1^). *Staphylococcus epidermidis* ATCC-14990 and *C. acnes* ATCC-11827 were incubated in a 1:1 ratio one day before *S. aureus* ATCC-25923. Each 3D skin model was inoculated in triplicate and then incubated at 37 °C in 5% CO^2^ at > 95% RH for 24 h. Then 10 μL of each test formulations were applied to the inoculated 3D skin models. A set of 3 inserts were also treated with 10 μL of a gel without preservatives and any other active ingredient. Tested formulations are reported in Table 1.

**Table 1:** List of formulations [C1-C12] containing preservatives used in this work.

INCI name	C1	C2	C3	C4	C5	C6	C7	C8	C9	C10	C11
AQUA	96.70	97.70	96.27	96.35	96.35	97.50	97.00	96.40	96.70		
Sodium benzoate	0.30				0.15						
Phenoxyethanol, ethylhexylglycerin	1.00										
Gluconolactone, sodium benzoate											
Phenoxyethanol											
Hydroxyacetophenone		0.30	0.30			0.30				0.30	
Phenylpropanol, propanediol, caprylyl glycol, tocopherol		1.00	1.00			1.00					
Sodium anisate				0.15							
1,2-Hexanediol				1.50	1.50			1.50		1.50	
Disodium EDTA						0.20					
AQUA, tetrasodium glutamate diacetate			0.43								
Benzyl alcohol, benzoic acid, dehydroacetic acid							1.00				
O-cymen-5-ol								0.10			
PPG-3 benzyl ether myristate								1.00			
1,2-Hexanediol, caprylyl glycol, tropolone									1.00		
Levulinic acid, sodium levulinate, glycerin									0.30		
Potassium sorbate, sodium benzoate											1.50
Ammonium acryloyldimethyltaurate/vp copolymer	2.00	2.00	1.00	2.00	2.00	2.00	1.00	2.00	1.00	2.00	2.00

Once obtained a gel, the different combinations of preservatives have been left in contact with inserts for 3 h and then two 4 mm biopsy samples were aseptically removed from each inoculated 3D skin model. For the microbial assay, the biopsy was immediately suspended in Dey-Engley Neutralising Broth and serial dilutions were made in phosphate buffer, and the appropriate dilutions were plated onto plate count agar specific for each strain. Cell growth was calculated as Log10 CFU/cm^2^ and also the % of inhibition was derived.

A 4 mm biopsy punch was also aseptically removed for qRT-PCR analysis. Total RNA was extracted by mean of Tri Reagent (Sigma Aldrich, Milan, Italy) as reported by Chomczynski and Mackey^18^. cDNA was synthesized from 2 μg RNA template using the Prime script RT reagent kit (Takara, Japan) according to manufacturer instruction using a thermal cycler (Stratagene Mx3000P Real Time PCR System, Agilent Technologies Italia S.p.A., Milan, Italy). Obtained cDNA was amplified and detected with the same instrument using the following Taqman gene expression assays: HS00187320_M1 (HDAC3) and Hs999999 m1 (GAPDH, human glyceraldehyde-3-phosphate dehydrogenase). Human GAPDH was used as the housekeeping gene. PCR amplifications were carried out on 40 ng of cDNA in a 20 μl of the final volume. Following PCR conditions were used: 50 °C for 2 min and 95 °C for 10 min followed by 40 amplification cycles (95 °C). Amplification was carried out in triplicate using, in particular, 10 μl of 2 × Premix Ex Taq (Takara, Japan), 1 μl of 20 × TaqMan gene expression assay, 0.4 μl of RoX Reference Dye II (Takara, Japan9, and 1 or 4 μl of DNA.

2^−ΔΔCt^ method^19^ was used to assess the relative abundance of the expression of analyzed genes.

### Statistical analysis

Statistically significant differences were obtained by t-tests and one-way analysis of variance (ANOVA) tests for independent samples corrected by using Tukey test. All datasets were normally distributed. Analyses were performed with GraphPad Prism 7.0 (GraphPad Software, Inc., San Diego, CA). Differences between groups were considered significant at a P-value < 0.05.

## Results and discussion

In the present work, different preservative combinations commonly used in cosmetics are evaluated in vitro for their activity on skin resident microflora by serial dilution on plate count agar (Table 2).

**Table 2:** Activity of the different combination of preservative tested on growth dynamic. Antimicrobial activity is expressed as Log10 CFU/cm^2^.

	Log10 CFU/cm^2^		Log10 CFU/cm^2^		Log10 CFU/cm^2^	
	*C. acnes*	sd	*S. aureus*	sd	*S. epidermidis*	sd
Untreated	10.02	0.02	7.67	0.03	6.92	0.13
C1	9.58	0.04	6.21	0.04	7.06	0.06
C2	8.67	0.19	5.83	0.13	6.85	0.14
C3	9.02	0.08	5.95	0.10	6.92	0.03
C4	9.63	0.04	5.97	0.07	6.85	0.02
C5	10.12	0.05	6.80	0.18	6.26	0.08
C6	9.74	0.13	6.23	0.05	7.20	0.10
C7	9.75	0.02	5.98	0.15	7.41	0.04
C8	10.16	0.06	5.77	0.07	5.86	0.09
C9	10.16	0.08	5.59	0.04	5.62	0.03
C10	10.65	0.04	5.06	0.08	6.61	0.03
C11	10.01	0.11	6.24	0.04	7.07	0.09

Tested preservatives differently influenced the growth dynamic of target bacteria (Table 3).

**Table 3:** Activity of the different combination of preservatives tested on growth dynamic expressed as % of inhibition.

	*C. acnes*	*S. aureus*	*S. epidermidis*
C1	+	++	−
C2	++	+++	−
C3	++	+++	−
C4	+	+++	−
C5	−	++	+
C6	+	++	−
C7	+	+++	−
C8	−	+++	++
C9	−	+++	++
C10	−	+++	+
C11	−	++	−

+++ strongly inhibited [< 75%]; ++. moderately inhibited [90–80%]; +, weakly inhibited [98–91%]; −, no inhibition.

The combinations of preservatives C2 (hydroxyacetophenone,phenylpropanol, propanediol, caprylyl glycol, tocopherol) and C3 (hydroxyacetophenone,phenylpropanol, propanediol, caprylyl glycol, tocopherol, and tetrasodium glutamate diacetate) act moderately inhibiting *C. acnes* and strongly *S. aureus* without simultaneously inhibiting the growth of *S. epidermidis*. The use of these combinations of preservatives could be advantageous for use in topical products for skin and scalp where it is necessary to restore a pre-existing condition of microbial dysbiosis.

The combinations of preservatives C1 (sodium benzoate phenoxyethanol, ethylhexylglycerin), C4 (sodium anisate, 1,2-hexanedio), C6 (hydroxyacetophenone, phenylpropanol, propanediol, caprylyl glycol, tocopherol, and disodium edta) and C7 (benzyl alcohol, benzoic acid and dehydroacetic acid), as they can slightly inhibit *C. acnes* and moderately *S. aureus* without simultaneously inhibiting the growth of *S. epidermidis* are suitable for use in topical products for skin and scalp in which it is necessary to preserve the eubiosis of the microbiota. This effect is also exerted by C11 (potassium sorbate, sodium benzoate9 although to a lesser extent.

Combination C10 (phenylpropanol, propanediol, caprylyl glycol, tocopherol and disodium edta) is able to strongly inhibit *S. aureus* without simultaneously inhibiting the growth of *C. acnes* and *S. epidermidis*. Its use could be advisable for use in topical products aimed at strongly counteract a dysbiosis caused by *S. aureus*.

The combinations of preservatives C5 (sodium benzoate and 1,2-hexanediol], C8 [1,2-hexanediol, o-cymen-5-ol and ppg-3 benzyl ether myristate) and C9 (1,2-hexanediol, caprylyl glycol, tropolone, levulinic acid, sodium levulinate, glycerin) strongly influenced also the growth dynamic of *S. epidermidis* and for this effect are not advisable for use in topical products aiming at restoring or maintain the skin microflora.

**Figure 1:**
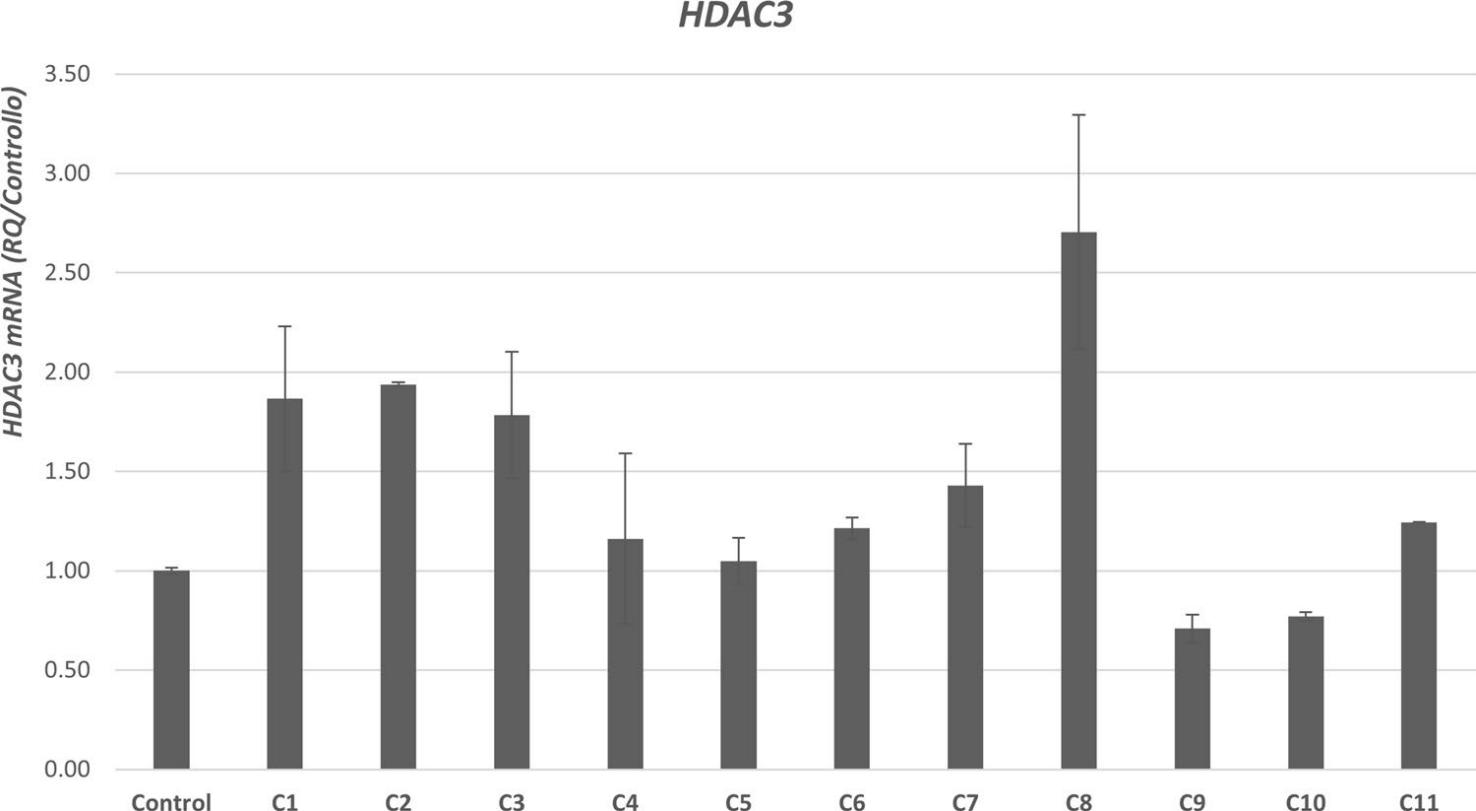
mRNA expression of histone-deacetylase-3 [HDAC3]. Histone deacetylase 3 **(**HDAC3) expression in control vs different (C1-C11) (Table 1) preservatives combinations. mRNA levels of HDAC3 detected by RT-PCR plus error bars.

In line with the effects of tested preservatives on the growth dynamic of main skin resident bacteria, through qRT-PCR we also highlighted a significant (p < 0.005) increase of the expression of HDAC3 by all the combinations of preservatives except for C4, C5, C9, and C10 (Fig. 1).

A central role in orchestrating host-microbiota interactions has been recently attributed to histone deacetylases (HDACs)^20^. In particular, HDAC3 is a key mediator in maintaining the integrity and function of several human organs including the gut and skin^16,17^. *Cutibacterium acnes*, as an example, has been reported to affect cutaneous inflammation through the epigenetic mechanism of HDAC activity^14^. HDAC3 also mediates allergic skin inflammation by regulating the expression of monocyte chemoattractant protein-1 (MCP1)^21^.

In presence of commensal "beneficial" bacteria that promote eubiosis HDAC3 plays a key role in the relationship between microbiota and inflammation^22^. Therefore some bacterial species, usually unbeneficial, can be effectively overpopulated when HDAC3 is under-expressed. Most important, HDAC is sensitive to environmental and extrinsic factors including preservatives and biocides. For these reasons, HDAC reveals as a useful marker of how the skin regulates the relationship between beneficial microbiota and skin cellular functions to maintain eubiosis, and inhibition of HDAC could be representative of a damage/dysbiosis of the skin resident flora.

In healthy conditions skin maintains a stable resident microflora which also acts preventing the colonization and invasion by pathogens and modulating innate and adaptive immunity. Several extrinsic and intrinsic factors could lead to a situation of dysbiosis, including cosmetic ingredients.

Preservatives commonly used in topical formulation possess a well-known antibacterial activity on major pathogens such a *S. aureus* and *Escherichia coli*; however, their effects on skin resident flora have been poorly investigated. This is a “burning” issue considering that the dynamics of the skin microbiome are fundamental for skin health. Wang and collaborator^23^ studied for the first time the effect of preservatives on facial skin microflora.

Here we investigated the effect of twelve different preservative combinations on the growth of the main three skin‐resident bacteria to have a better understanding of their effect on the dynamics of skin‐resident bacteria and specifically on eubiotic or dysbiotic conditions.

The genera of stable Gram-positive bacteria [G +], *Cutibacterium*, and *Staphylococcus*, are retained as fundamental components of skin microbiota^24^. In particular, *C. acnes*,* S. epidermidis*, and *S. aureus* act usually as commensal bacteria since they are harmless when the skin is healthy and are in a mutualistic relationship with the cutaneous system^25^. *Staphylococcus epidermidis*, is the most beneficial one. Among other activities, it has the useful ability to inhibit the growth of pathogenic strain of *S. aureus* and *S. epidermidis* forming biofilms^26^ via secretion of specific bacteriocins. *Staphylococcus epidermidis* and *S. aureus* are in strict relation to each other and a balanced ratio between these two is supposed to be beneficial for the skin microbiome^27^.

*Staphylococcus aureus* is the most “virulent” cousin of *S. epidermidis* since it has been related, for example, to some skin conditions in which an unbalancing of skin microbiota has been reported such as atopic dermatitis^28^.

Beyond its role in maintaining the homeostasis of the skin’s microbiome, *C. acnes* may become “pathogenic” for the skin. It has long been implicated in the pathogenesis of acne^29^. An unbalancing in the bacterial population, including *C. acnes* and *S. aureus* has been also recently reported in other skin conditions such as seborrheic dermatitis^30^, psoriasis, and rosacea^31^, and alopecia^32–35^.

## Conclusions

In this study, eleven different preservative combinations were tested for their effects on skin resident microflora’s dynamic in a 3D skin models. Tested combinations differently influenced the growth dynamic of target bacteria. Combinations containing hydroxyacetophenone, phenylpropanol, propanediol, caprylyl glycol, tocopherol, and tetrasodium glutamate diacetate resulted as the most suitable for restoring a pre-existing dysbiosis since they act moderately inhibiting *C. acnes* and strongly *S. aureus* without simultaneously inhibiting the growth of *S. epidermidis*.

Combinations C2, C3, and C10 resulted as the best suitable for restoring a pre-existing dysbiosis since they act moderately inhibiting *C. acnes* and strongly *S. aureus* without simultaneously inhibiting the growth of *S. epidermidis*. Combinations C1, C4, C6, and C7 resulted as the best suitable for use in topical products for skin and scalp in which it is necessary to preserve the eubiosis of the microbiota. All the combinations except for C4, C5, C9, and C10 were also able to increase HDAC3 expression.

Taking together these data highlight the role of preservatives of skin resident microflora dynamics and could provide a reference for correctly choice preservatives and dosage in cosmetic formulations to preserve or restore homeostasis of skin microorganisms.
